# Endoscopic management of patients with familial adenomatous polyposis after prophylactic colectomy or restorative proctocolectomy – systematic review of the literature

**DOI:** 10.2478/raon-2024-0029

**Published:** 2024-06-11

**Authors:** Aleksandar Gavric, Liseth Rivero Sanchez, Angelo Brunori, Raquel Bravo, Francesc Balaguer, Maria Pellisé

**Affiliations:** Department of Gastroenterology and Hepatology, University Medical Centre Ljubljana, Slovenia; Department of Gastroenterology, Hospital Clinic de Barcelona, Barcelona, Spain; Institut d’Investigacions Biomediques August Pi I Sunyer (IDIBAPS), Barcelona, Spain; Center for Biomedical Research in the Hepatic and Digestive Diseases Network (CIBERehd), Barcelona, Spain; Surgery Department, Hospital Clinic de Barcelona, Barcelona, Spain

**Keywords:** familial adenomatous polyposis, ileorectal anastomosis, ileal pouch-anal anastomosis

## Abstract

**Background:**

Patients with familial adenomatous polyposis (FAP) develop early colorectal adenomas and if left untreated, progression to cancer is an inevitable event. Prophylactic surgery does not prevent further development of cancer in the rectal remnant, rectal cuff in patients with ileal pouch anal anastomosis (IPAA) and even on the ileal mucosa of the pouch body. The aim of this review is to assess long-term rates of cancer and adenoma development in patients with FAP after prophylactic surgery and to summarise current recommendations for endoscopic management and surveillance of these patients.

**Materials and methods:**

A systematic literature search of studies from January 1946 through to June 2023 was conducted using the PRISMA checklist. The electronic database PubMed was searched.

**Results:**

Fifty-four papers involving 5010 patients were reviewed. Cancer rate in the rectal remnant was 8.8–16.7% in the western population and 37% in the eastern population. The cumulative risk of cancer 30 years after surgery was 24%. Mortality due to cancer in the rectal remnant is 1.1–11.1% with a 5-year survival rate of 55%. The adenoma rate after primary IPAA was 9.4–85% with a cumulative risk of 85% 20 years after surgery and a cumulative risk of 12% for advanced adenomas 10 years after surgery. Cumulative risk for adenomas after ileorectal anastomosis (IRA) was 85% after 5 and 100% after 10 years. Adenomas developed more frequently after stapled (33.9–57%) compared to hand-sewn (0–33%) anastomosis. We identified reports of 45 cancers in patients after IPAA of which 30 were in the pouch body and 15 in the rectal cuff or at the anastomosis.

**Conclusions:**

There was a significant incidence of cancer and adenomas in the rectal remnant and ileal pouch of FAP patients during the long-term follow-up. Regular endoscopic surveillance is recommended, not only in IRA patients, but also in pouch patients after proctocolectomy.

## Introduction

Familial adenomatous polyposis (FAP) is an autosomal dominant inherited disease caused by pathogenic variants in the adenomatous polyposis coli (APC) gene^1^ with reported incidence of one in 8,000 to 12,000 live births.^2^ The main hallmark of the disease is the presence of multiple colorectal adenomas, leading to a 100% lifetime risk of developing cancer if the colon remains in situ.^3^ To prevent the development of cancer, prophylactic colectomy or proctocolectomy is performed when the adenoma burden cannot be managed endoscopically or at the age of 18–25 years old. The following types of surgery are available^4^: total colectomy with ileorectal anastomosis (IRA) or ileosigmoid anastomosis (ISA); proctocolectomy with/without mucosectomy and stapled ileal pouch-anal anastomosis (IPAA) or hand-sewn IPAA; and total proctocolectomy with end ileostomy. Until restorative proctocolectomy with IPAA and pouch reconstruction was described in the 1970s, colectomy with IRA or end ileostomy was the only surgical prophylactic procedure available and was associated to a considerable high CRC incidence and mortality.^5^ After this, proctocolectomy with pouch reconstruction (IPAA) was the technique of choice in patients with a high adenoma burden and was sought to eliminate the risk of CRC in FAP patients. However, since the first report of pouch cancer in 1994^6^, there has been a substantial increase in published literature reporting rates of adenoma and cancer development after primary IPAA. The development of adenomas along life in remnant rectal mucosa is a natural phenomenon in this population. Long live periodical surveillance with rectoscopies is widely recommended in international guidelines as shown in Table 1.^4,7,8,9,10^ As there are no randomised trials comparing endoscopic surveillance and management strategies for FAP patients with IRA and IPAA, we aimed to systematically evaluate adenoma and cancer development after prophylactic surgery, define potential risk factors and to summarise endoscopic practices from published series.

**TABLE 1.** Summary of recommendations from the international guidelines

**First author and publication date (ref.)**	**Endoscopic surveillance – patients with IRA**	**Indications for secondary proctectomy patients with IRA**	**Endoscopic surveillance – patients with IPAA**
Vasen et al., 2008^7^	Every 3 to 6 months	Multiple large adenomas (> 5 mm) Adenomas with dysplasia	Every 6 to 12 months
Balmaña et al., 2013, ESMO^8^	Every 12 months	No recommendations	Every 12 months
Stoffel et al., 2015, ASCO^9^	Every 6 to 12 months	No recommendations	Every 6 months to 5 years (Intervals should be determined on a case-by-case basis and may be even shorter than 1 year for some individuals)
Sygnal et al., 2015, ACG^10^	Every 12 months	No recommendations	Every 12 months
Herzig et al., 2017, ASCRS4	Every 12 months	No recommendations	Every 12 months
Van Leerdam ME et al., 2019, ESGE^53^	Every 12 to 24 months	No recommendations	Every 12 to 24 months
Yang J et al., 2020, ASGE^54^	6 months after surgery with 6 to 12 months further surveillance interval		12 months after surgery with 12 to 24 months further surveillance interval. 6 months if advance adenoma

ACG = American College of Gastroenterology; ASCO = American Society of Clinical Oncology; ASCRS = American Society of Colon and Rectal Surgeons; ASGE = American Society for Gastrointestinal Endoscopy; ESGE = European Society of Gastrointestinal Endoscopy; ESMO = European Society for Medical Oncology; IPAA = ileal pouch anal anastomosis; IRA = ileorectal anastomosis

## Materials and methods

Our review is reported according to the PRISMA guidelines.^11^

### Search strategy

We searched PUBMED from inception to June 2023 to identify studies evaluating long-term adenoma and cancer development in patients with FAP after prophylactic surgery. Deduplication was performed using Zotero software.^12^ Reference lists of included studies were hand-searched for additional relevant studies. The search was limited to studies, published in English. We used the following keywords: “FAP”, “IRA”, “IPAA”, “familial adenomatous polyposis” and “proctocolectomy”.

### Inclusion criteria

We included single-or multicentre retrospective cohort studies, prospective cohort studies and retrospective analyses of polyposis registries. Due to the rarity of the events, we only considered case reports for inclusion when summarising reports on cancers after primary IPAA. Only the most recent series from the same institution or polyposis registry were included in the analysis, as some research groups regularly publish retrospective analyses of their cohorts or polyposis registries. Full-text screening and data extraction were performed by a single researcher (AG). Manuscripts of three case reports could not be obtained, data were summarised from the two review articles.^13,14^

## Results

### Studies identified

**FIGURE 1.**
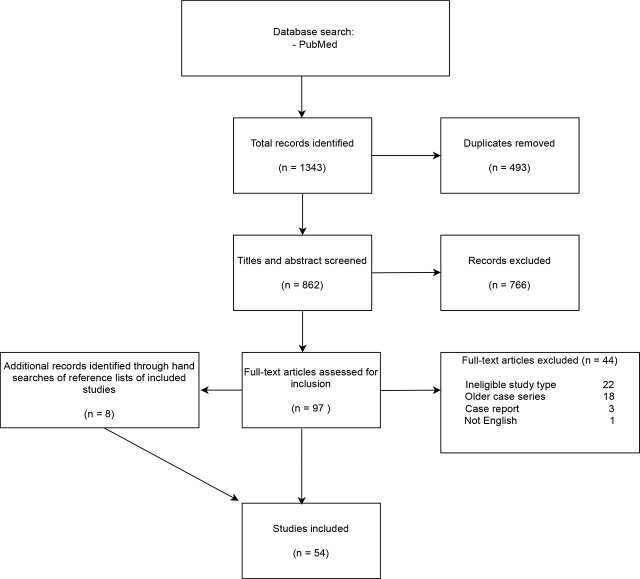
Flowchart of the systematic review according to the Preferred Reporting Items for Systematic Reviews (PRISMA) schema.

Of 97 full-text articles screened for eligibility (Figure 1), 46 met our inclusion criteria. A further 8 articles were identified by hand searching the reference lists of the included studies (6 case reports, 1 retrospective cohort, 1 polyposis registry analysis). We included 22 retrospective analyses, 14 case reports (carcinoma development after primary IPAA), 15 retrospective analyses of prospectively maintained polyposis registries and 3 prospective cohort studies. Only 5 studies were multicentre and 1 was bi-centre. The studies were published between 1994 and 2023. The studies included between 1 and 925 patients. A total of 5010 patients were included in the review. Summary characteristics of the included studies are shown in Table 2.

**TABLE 2.** Characteristics of included studies

**First author and publication date (ref.)**	**No. of patients**	**Country**	**Setting**	**Study design**	**Surgery performed (period)**	**Study population**
Aelvoet *et al.*, 2023^55^	144 (111 IPAA, 33 ileostomy)	The Netherlands	Single	Cohort/Retrospective	/	IPAA, ileostomy
Tatsuta *et al.*, 2023^56^	65 (22 IRA, 20 IPAA)	Japan	Single	Cohort/Retrospective	1976–2022	IRA, IPAA
Anele *et al.*, 2022^57^	199 (199 IRA)	United Kingdom	Single	Cohort/Retrospective	1990–2017	IRA
Colletti *et al.*, 2022^58^	715 (715 IRA)	Italy	Multicentre	Retrospective analysis of the Registry	1977–2021	IRA
Pasquer *et al.*, 2021^59^	289 (197 IRA, 92 IPAA)	France	Multicentre	Retrospective analysis of the Registry	1965–2015	IRA, IPAA
Ardoino *et al.*, 2020^60^	925 (585 IRA, 340 IPAA)	Italy	Multicenter	Retrospective analysis of the Registry	1947–2015	IRA, IPAA
Tajika *et al.*, 2019^16^	47 (14 IRA, 25 IPAA, 8 ileostomy)	Japan	Single	Cohort/Retrospective	1965–2017	IRA, IPAA and ileostomy
Ganschow *et al.*, 2018^61^	192	Germany	Singe	Cohort/Prospective and retrospective analysis of Polyposis Registry	Endoscopy data collected during 2010–2013	IPAA
Kariv *et al.*, 2017^62^	45	Israel	Single	Cohort/Retrospective	1986–2013	IPAA
Patel *et al.*, 2016^42^	21 (6 IRA, 5 IPAA, 10 intact colon)	Indianapolis, USA	Single	Cohort/Retrospective	Endoscopies performed between 2004–2016	IRA, IPAA and intact colon
Walsh *et al.*, 2016^63^	1	Ireland	Single	Case report	1987	IPAA - cancer
Maehata *et al.*, 2015^20^	27	Japan	Single	Cohort/Retrospective	1990–2004	IRA
Ganschow *et al.*, 2015^50^	100; 50 hand-sewn and 50 stapled anastomoses	Germany	Single	Cohort/Prospective	?	Hand-sewn *vs*. stapled anastomosis
Goldstein *et al.*, 2015^63^	59	Israel	Single	Cohort/Retrospective	1986–2013	IPAA
Zahid *et al.*, 2015^64^	27	Australia	Single	Cohort/Retrospective	1984–2011	IPAA
Kennedy *et al.*, 2014^65^	95; 85 hand-sewn and 1 stapled anastomosis	Rochester, Mayo Clinic, USA	Single	Cohort/Retrospective	1987–2011	IPAA
Koskenvuo *et al.*, 2013^22^	140	Finland	Single	Cohort/Retrospective	1963–2012	IRA
Pommaret *et al*., 2013^35^	118	France	Single	Cohort/Retrospective	/	IPAA and IRA
Boostrom *et al*., 2013^66^	117	Rochester, Mayo Clinic, USA	Single	Cohort/Retrospective	1972–2007	IPAA
Ozdemir *et al*., 2013^37^	260; 86 hand-sewn and 175 stapled anastomoses	Cleveland, USA	Single	Analysis of polyposis registry	1983–2010	Hand-sewn *vs*. stapled anastomosis
Wasmuth *et al*., 2013^67^	61; 39 hand-sewn with mucosectomy and 22 without of which 15 were stapled and 7 hand-sewn anastomoses	Norway	Multicenter	Analysis of polyposis registry	1986–2008	IPAA (mucosectomy *vs.* no-mucosectomy)
Yan *et al*., 2012^68^	42 (33 IPAA; 6 IRA ?)	China	Single	Cohort/Retrospective	1988–2008	IPAA and IRA
Makni *et al*., 2012^69^	1	Tunisia	Single	Case report	1996	IPAA - cancer
Tonelli *et al.*, 2012^51^	69	Italy	Single	Cohort/Prospective data collection	1984–2008	IPAA
von Roon *et al*., 2011^70^	140; 44 hand-sewn and 76 stapled anastomoses	UK	Single	Retrospective analysis of St. Mark’s Hospital Polyposis Registry	1978–2007	Hand-sewn *vs*. stapled anastomosis
Banasiewicz *et al*., 2011^32^	165	Poland	Bicenter	Bicenter/Retrospective analysis	1985–2009 operated, Clinical data from endoscopy FUP between 2004–2009	IPAA
Booij *et al*., 2010^18^	43 (34 IRA)	The Netherlands	Single	Cohort/Retrospective	1977–2005	IRA and IPAA
Sinha *et al*., 2010^26^	427	UK	Single	Retrospective analysis of St. Mark’s Hospital Polyposis Registry	1990–2008	IRA
Ault *et al*., 2009^71^	2	Los Angeles, USA	Single	Case series	1990, 1993	IPAA - cancer
Nieuwenhuis *et al*., 2009^27^	475	Denmark, Finland, Sweden, Netherlands	Multicenter	Analysis of polyposis registry	/	IRA
Yamaguchi *et al*., 2009^25^	59	Japan	Single	Cohort/Retrospective	1962–2007	IRA
Friederich *et al*., 2008^31^	212; 71 hand-sewn with mucosectomy and 115 stapled anastomoses	The Netherlands	Single	Analysis of National Polyposis Registry	1985–2005	IPAA
Campos *et al*., 2008^19^	36	Brasil	Single	Cohort/Retrospective	1977–2006	IRA and IPAA
Bullow *et al*., 2008^24^	776; 576 operated in pre-pouch period and 200 in pouch period starting in 1990	Denmark, Finland, Sweden, Netherlands	Multicenter	Analysis of polyposis registry	1950–2006	IRA
Gleeson *et al*., 2008^30^	16	Rochester, Mayo Clinic, USA	Single	Cohort/Retrospective analysis	1964–2003(Analysis of endoscopies between 1992–2006)	IPAA and IRA
Lee *et al*., 2008^72^	1	Korea	Single	Case report	1998	IPAA - cancer
Linehan *et al*., 2007^73^	1	Ireland	Single	Case report	1997	IPAA - cancer
Valanzano *et al*., 2007^28^	25	Italy	Single	Cohort/Prospective	1986–2004	IRA
Moussata *et al*., 2007^17^	21	France	Single	Cohort/Retrospective	/	IPAA and IRA
Ulas *et al*., 2006^74^	1	Turkey	Single	Case report	1993	IPAA - cancer
Campos *et al*., 2005^19^	1	Brazil	Single	Case report	/	IPAA - cancer
Groves *et al*., 2005^34^	60	UK	Single	Retrospective analysis of St. Mark’s Hospital Polyposis Registry	/	IPAA
Vroueraets *et al*., 2004^75^	2	The Netherlands	Single	Case report	1990, 1991	IPAA – cancer
Ooi *et al*., 2003^36^	2	Cleveland, USA	Single	Case report	/	IPAA – cancer
Church *et al*., 2003^38^	197; 62 operated in pre-pouch period and 135 in pouch period starting in 1983	Cleveland, USA	Single	Analysis of polyposis registry	1950–1999	IRA
Cherki *et al*., 2003^76^	1	France	Single	Case report	/	IPAA - cancer
Thompson-Fawcett *et al*., 2001^77^	33	Canada	Single	Cohort/Prospective	/	IPAA
Church *et al*., 2001^15^	213 (165 IRA)	Cleveland, USA	Single	Analysis of polyposis registry	/	IRA and IPAA
Brown *et al*., 2001^78^	1	Singapore	Single	Case report	/	IPAA - cancer
Bertario *et al*., 2000^23^	371	Italy	Multicenter	Retrospective analysis of Hereditary tumor registry	1955–1997	IRA
Vuilleumier *et al*., 2000^79^	1	UK	Single	Case report	1990	IPAA - cancer
Jenner *et al*., 1998^21^	55	Australia	Single	Analysis of polyposis registry	?–1994	IRA
Bassuini *et al*., 1996^80^	1	UK	Single	Case report	1991	IPAA - cancer
Hoehner *et al*., 199^46^	1	Iowa, USA	Single	Case report	/	IPAA - cancer

FUP = follow up; IPAA = ileal pouch anal anastomosis; IRA = ileorectal anastomosis

### Total colectomy with ileorectal anastomosis

#### Adenomas

Five studies described the rate of adenoma development in the residual rectum (Supplementary Table 1). In 8 studies that analysed the frequency of secondary proctectomy due to endoscopically unmanageable polyposis, the rate of proctectomy ranged from 3.7% to 35%.^15^ Five studies described adenoma evaluated in the neoterminal ileum (Table 3), with a high variance in reported rates from 0%^16^ to 47.6% in patients followed-up for median of > 20 years^17^ in one study including a paediatric cohort^18^, 2 patients required resection of the terminal ileum and construction of a new IRA, one due to low grade dysplasia (LGD) and one due to high grade dysplasia (HGD) adenoma.

**TABLE 3.** Rate of adenoma development in the neoterminal ileum in patients after ileorectal anastomosis (IRA) and ileal pouch anal anastomosis (IPAA)

**First author and publication date (ref.)**	**Adenomas in the neoterminal ileum – after primary IPAA; n (%)**	**Cumulative risk for development of neoterminal adenomas**	**Years since surgery**	**Risk factor for adenomas in neoterminal ileum**	**Rate of adenomas in the neoterminal ileum – after primary IRA; n (%)**	**Years since surgery**
Tajika *et al*., 2019^16^	4/24 (16.7)	4.4% at 20 years and 36% at 30 years after primary surgery	23.1 ± 5.8		0/14 (0.0)	
Boostrom *et al*., 2013^66^	4/33 polyps (12.0)					
Pommaretet *et al*., 2013^35^	9/118 (6.5)			Presence of pouch adenomas (OR, 2.16, *P* = 0.007)		
Booij *et al.*, 2010^18^					5/34 (14.7) 2 patients had resection of neo-terminal ileum, one due to LGD and other due to HGD adenoma.	
Gleeson *et al*., 2008^30^	3/13 (23.1)		Median 6.5 (0–15)		4/16 (25.0)	Median 12 (1–29)
Moussata *et al*., 2007^17^			Mean 17.6 +-7.8(6–35) Mean from colectomy to diagnosis: 16.4+-8.5 (5–30)		10/21 (47.6) of which 2 were advanced adenomas.	
Groves *et al*., 2005^34^	2/20 (10.0)		6 (1–14)		1/47 (2.0%)	12 (0–39)
Thompson-Fawcett *et al*., 2001^77^	1/24 (4.2)		Median 7 (1–19)			

HGD = high grade dysplasia; LGD = low grade dysplasia

#### Rectal cancer

The reported rate of cancer in the rectal remnant (Table 4) after primary IRA is 8.8%^18^ to 16.7%19 with a median follow-up from surgery^19^ of 91.1 months (3–557 months). However, studies from Japan report higher rates of up to 37%^20^, but this is due to the inclusion of *in situ carcinoma* in the cancer definition. The same study had the longest median follow-up of 21.1 years (3–35). On the other hand, a small cohort of 21 patients from France reported zero cases of cancer during a median follow-up of 8.4 years. Jenner *et al*.^21^ only included patients with a confirmed mutation. Five studies reported a cumulative incidence of rectal cancer ranging from 3%^22^ to 17.2%^19^ at 5 years, 7.7%^23^ to 24.1%^19^ at 10 years, 11%^22^ to 23%^23^ at 20 years, and 24%^22^ at 30 years after the primary IRA. In one of the largest studies^24^, which analysed data from 4 national registries and 776 patients, the 10-year cumulative risk of residual rectal cancer was 4.4% (95% CI, 2.6–6.2) for patients who underwent surgery before 1990 and only 2.5% (0–5.5) after the 1990. Only one study reported the time from surgery to cancer diagnosis (median 102 months [1–26 years])^23^; other studies reported follow-up time from surgery, but did not clearly define when follow-up started nor the surveillance regime. Five studies reported mortality ranging from 1.6%^23^ to 11.1%^20^ in which 3 out of 27 patients died from cancer in the rectal remnant. Only one of two studies that examined long-term survival after diagnosis of residual rectal cancer reported a 5-year survival rate of 55%.^22^ In a study from Japan, 5-year survival was 94%^25^, but the excellent survival was explained by the inclusion of carcinoma in situ despite the exact proportion of these was not given.

**TABLE 4.** Patient characteristics and rate of rectal remnant cancer rate in patients after ileorectal anastomosis (IRA)

**First author and publication date**	**Proportion of man; n / (%)**	**APC mutation Underwent n/(%); Positive in; n/(%)**	**Follow-up (years/months) since surgery**	**Years since surgery to cancer diagnosis**	**Age at surgery**	**Age at cancer diagnosis**	**Rectal remnant cancer rate; n/(%)**	**Cumulative risk for rectal cancer**	**Rectal cancer mortality**
Colletti *et al*., 2022^58^	57.4%	93.6% /	/	Median of 13 years	/	/	47 / 715 (6.57)	/	14/47 (29.8%) at median follow up of 13 years.
Pasquer *et al*., 2021^59^	95 (48.2)	/	/	/	/	/	12 / (6.1); 1 was metastatic, 2 were resected endoscopically, 10 surgically	/	/
Maehata *et al*., 2015^20^	16 (59.3)	21 (77.8) 14 (66.7)	21.1 (3–35)	/	Median 27 years (9–66)	/	10/27 (37.0); 6/10 cancers were TisN0M0	8% at 10 years; 19% at 20 years; 57% at 30 years	3/27 (11.1)
Koskenvuo *et al*., 2013^22^	59 (42.1)	/	Median 15 years (0–44)	/	Mean 36 years (18–71)	Cumulative risk 2% at 40 years age; 7% at 50; 13% at 60 years age and 16 % at 70 years age.	18/140 (13%)	3% at 5 years; 4% at 10 years; 11% at 20 years; 24% at 30 years after IRA	10/140 (7%); 5-year survival 55%. Cumulative risk for death due to rectal cancer after IRA: 2% at 5 years, 3% at 10 years and 9% at 30 years.
Booij *et al*., 2010^18^	19 (44.2)	/	/	/	Median 16 (7–25)	/	3/34 (8.8)	/	2/34 (5.8)
Sinha *et al*., 2010^26^	232 (54.3)	/ 311/427 (72.8)	Median 15 years (7–25)	/	Median 21 years (11–67)	/	48/427 (11.2%)	/	/
Yamaguchi *et al*., 2009^25^	35 (59.3)	/	Median 8.9 years	/	Median 30 years (13–65)	/	17/59 (30%)	/	5-year survival 94%; 10-year survival 94%.
Nieuwenhuis *et al*., 2009^27^	/	/	/	/	/	/	/	3.7% for group 1; 9.3% for group 2; 8.3% for group 3.%	/
Campos *et al*., 2008^19^	/	/	91.1 (3–557)	/	Mean 45.8 years	Mean 50.6 years	6/36 (16.7)	17.2% at 5 years; 24.1% at 10 years; 43.1% after 15 years	/
Gleeson et al., 2008^30^	/	/	FUP initiated median 12 (1–29) years after surgery	/	/	40 and 59 years.	2/16 (12.5)	/	/
Bullow *et al*., 2008^24^	401 (51.7)	/	Median 7 years (0–13). Patients were operated between 1950–2006		Median 27 (7–75)	/	60/776 (7.7%) (56/576; 10% and 4/200; 2%)	10-year cumulative risk 4.4% [95% CI 2.6–6.2] in pre-pouch era; 10-year cumulative risk 2.5% [95% CI 0–5.5] in pouch era;	/
Moussata *et al*., 2007^17^ They only watched ileal muocas above the IRA	10 (47.6)	21/21 (100.0) 14/21 (66.7)	Mean 8.4 years ± 5 since colectomy	/	/	/	0/21 (0.0)	/	/
Church *et al*., 2003^38^	92 / (46.7)	/	Pre-pouch era: 212 months (IQR 148 months); Pouch era: 60 months (IQR 80 months)	/	Median age 23 years (IQR 15.5 years pre-pouch and 17 years pouch)	/	8 (12.9%) in the pre-pouch era and 0 in pouch era.	/	/
Bertario *et al*., 2000^23^	206/371 (55.5)	297/371 (80.1) 200/297 (67.3)	Median 81 months	Median 102 months (1–26 years)	Mean 32 years	/	27/371 (7.3)	10 years – 7.7% 15 years – 13.1 % 20 years – 23.0%	6/371 (1.6)
Jenner *et al*., 1998^21^	25/55 (45.0)	55/(100.0)	Median 10 (1–31)	/	Mean age 30 (13–62)	Median 41	7/55 (12.7)	/	/

Colonic phenotype divided in 3 groups: (Group 1 - <100 polyps and mutation in codons 1–157, 312–412 and 1596–2843; Group 2 Hundred of polyps and mutation in codons 158–311, 413–1249 and 1465–1595; Group 3 Thousand of polyps and mutation in codon 125

APC = adenomatous polyposis coli; FUP = follow up

#### Risk factors for progressive phenotype of rectal remnant

Eleven studies reported nine risk factors predictive of the progressive rectal residual phenotype (Supplementary Table 2). Four studies analysed the genotype-phenotype relationship; The presence of a pathogenic variant between codons 1250–1464 was an independent risk factor for subsequent cancer development (HR 4.4 [1.3–15.0]^23^ and for the secondary proctectomy^26,27^ (HR 3.91 [1.45–10.51], *P* = 0.007). In a small study of 25 patients, all patients (n = 3) with carpeting rectal remnant polyposis had a pathogenic variant in codon 1309, but this was only descriptive data.^28^ An aggressive colonic phenotype with at least 500 polyps at time for surgery was identified as a risk factor in three studies (Supplementary Table 2). Two studies^15,25^ have identified > 20 rectal remnant polyps at the time of surgery or during the endoscopic surveillance^26^ as an independent risk factor for secondary proctectomy (HR 30.99 [9.57–100.32] *P* < 0.001), while in one study a cut-off of > 10 rectal adenomas^28^ was associated with a more aggressive phenotype, as these patients developed a mean of 9.29 rectal residual adenomas per patient per year compared with 0.67 adenomas per patient per year if they had < 5 rectal polyps at the time of surgery. Other potential risk factors included patient age at diagnosis of rectal residual cancer, time since surgery, presence of congenital hypertrophy of the retinal pigment epithelium, and presence of colon cancer at the time of primary surgery. APC site mutation, preoperative colon phenotype, presence of duodenal adenomas and rectal remnant phenotype on surveillance were not identified as risk factors for progressive rectal remnant disease phenotype only in one study.^20^

### Proctocolectomy with ileal-pouch anal anastomosis

#### Adenomas

Seventeen studies (Table 5) reported on the development of adenomas after IPAA, of which eight studies differentiated between the pouch body and the anastomosis, one study only reported the anastomotic adenoma rate, while in the remaining seven studies the authors did not precisely define the anatomical location of the adenomas. The median age of patients at the time of surgery ranged from 15.4 to 34.6 years, with a median follow-up from surgery of 5.4 years to a median of 21.6 years. The reported rate of adenoma in the pouch body ranged from 9.4%^29^ to 76.9%.^30^ The proportion of HGD histology among adenomas at the polyp level ranged from 5.9%^17^ to 53.2%.^31^ In one study, the proportion of advanced adenomas on a per-patient basis was 11.2%.^31^ The cumulative risk of adenoma development after primary IPAA was 12% and 58% at 5 and 20 years after the surgery respectively.^16^ According to the analysis from Poland^32^, 50% of all patients would develop LGD 15 years after the surgery, while HGD is estimated to be present in half of the patients 17.5 years after the surgery. Six studies analysed the rate of adenoma development in the neo terminal ileum, the proportion of patients with histologically confirmed adenoma varied from 4.2%^33^ to 23.1%^30^ with at a median follow-up from surgery of 6.5^34^ to 23.1 years.^16^ The cumulative risk of developing an adenoma in the neo terminal ileum was 4.4% at 20 years and increased to 36% at 30 years after the surgery as reported in the same study. The presence of pouch body adenomas was the only independent risk factor for the neo terminal ileum adenomas (OR, 2.16, *P* = 0.007).^35^

**TABLE 5.** Patient characteristics and rate of adenomas in patients after primary ileal pouch anal anastomosis (IPAA)

**First author and publication date**	**Sex (man); n (%)**	**APC mutation Underwent; n (%); Positive in; n (%)**	**Distinguish between pouch body and rectal cuff**	**Follow-up (months/years)**	**Time from surgery to first adenomas (years)**	**Age at surgery (years)**	**Rate of adenomas (≥ 1 polyp)**	**Size of adenomas, mm**	**Histology of adenomas; n (%)**	**Number of Adenomas**
Aelvoet AS *et al.*, 2023^55^	81 (56)	101 (91) 96 (86)	Yes	Median 152 (77–240)	15% at 5 years; 48% at 10 years; 85% at 20 years.	Median 24 (18–32)		Median 5 (3–15)	Tubular adenomas 31 (28%), Tubulovillous 26 (23%), Villous 5 (5%)	Prepouch ileum 4(2–13), Pouch body 20 (5–50), rectal cuff 6 (3–10)
Tajika *et al*., 2019^26^	16 (47.1)	/	Yes	Median 21.6 (3.7–8.8)	32 (35.9) of patients showed progression of pouch adenomas during FUP	Median 34.6 (17–52)	24/34 (70.6)	2–40 mm	6 advanced adenomas (25.0)	1–300
Ganschow *et al*., 2018^61^	100 (52.1)	133 (69.3)) ? / 133	No	Median 12.8 (9–17) for patients with pouch adenomas and (2.5–12.2) for patients without pouch adenomas;	32 (35.9) of patients showed progression of pouch adenomas during FUP	27.5 years (10.2–58.5)	90/192 (46.9) at a median of 8.5 years (0.9–25.1) after IPAA. 5 years after IPAA 84.9% patients free of adenoma; 15 years after 40.4% and 20 years after 21.9% patients were free of adenomas.	53/192 (58.9) ≤ 4 mm; 24/192 (26.7) 5 – 10 mm; 13/192 (14.4) ≥ 10 mm	Tubular adenomas in 69/192 (76.7); tubulovillous adenomas in 16/192 (17.8); villous in 5/192 (5.6)	46/192 (51.1) had < 4; 14/192 (15.6) 5–10; 30/192 (33.3) > 10 adenomas
Goldstein *et al*., 2015^63^	24 (41.0)		Yes	Mean 11.6 years +-14.6 years	Median adenoma free time interval since surgery; Cuff 10.8 years Pouch 16.9 years	Mean 30.8 years +-10.8 years	35/59 (59.0); - 20 isolated in cuff - 4 isolated in pouch body - 11 in pouch and body	/	All LGD	/
Zahid *et al*., 2015^19^	14 (51.8)		No	Mean 9.2 years	Median; 72 months (18–249)	Median 31 years (14–65)	12/27 (44.0)	/	Only 1 polyp HGD (< 99%)	/
Kennedy *et al*., 2014^66^	43 (45.0)		Watched only anastomosis	Mean 7.6 (0 – 24)		Mean 15.4 (4–20)	9/95 (9.4)			
Pommaretet *et al*., 2013^36^		110 / 139 92 / 110 (Cohort included IRA, ileostomy and IPAA patients but did not distinguish between).		/	Median 15 years	25 years (9−61 years)	57/118 (48.3)	> 10 mm:12	94% LGD; 6% HGD	1−4: 22 5−20: 18 > 20: 17
Boostrom *et al*., 2013^66^	52 (44.5)		Yes	125 months (25–423 months)	12.4 years (15–405 months)	26 years (4–60 years)	30/117 (25.6)	5.9 mm (2 mm to 20 mm)	22 LGD, 8 tubulovillous	/
Wasmuth *et al*., 2013^67^	34 (55.7)	/	Yes (body and anastomosis)		Cumulative rate of adenomas at 28 years 17% for mucosectomy group and 75% at 15 years in a group without mucosectomy (P < 0.0001)	20 (10–49)	Anastomosis: 4/39 (10.0) *vs.* 14/22 (64.0) (P < 0.0001) Pouch body: 8/39 *vs.* 6/22 (P 0.57)			
Tonelli *et al*., 2012^51^	/	45 (65%)	No	Median 133 months (12–288 months)	Mean 7 years (1–15 years)	33 years (17–63 years)	25 (36.0)	Mean 3 mm (1–40)	Adenomas, dysplasia not specified	Mean 8 (1–47)
Yan *et al*., 2012^68^	30 (71.5)	/	Yes	Median 7.2 (2.2–20)		29 (16–65)	At the anastomosis 6/33 (18.2)	/	/	/
Banasiewicz *et al*., 2011^33^	79 (47.9)	/	/	Endoscopies performed 2–19 years since surgery.	Mean 14 months to LGD; Mean 16 months to HGD. Estimated frequency LGD 15 years later 50% and for HGD 17.5 years later 50%.		21/165 (12.7)		LGD - 21/32 (65.6); HGD - 11/32 (34.4)	
Gleeson *et al*., 2008^31^	/	/	Yes	/	FUP began median 6.5 (0–15) after surgery	/	13/13 (100): 10/13 pouch body; 2/13 anastomosis; 3/13 ileum above anastomosis	< 5 mm	/	5–30
Friederich *et al*., 2008^32^	119 (56.0)	/	/	Mean 7.9 (0.4–20.3 years)	Cumulative risk of 16% at 5-years and 42.4% at 10 years for adenoma development. Cumulative risk of 12.8% at 10 years for advanced adenoma development.	Mean 30.0 years (10–62.6 years)	47/212 (35%)	/	/	/
Campos *et al*., 2008^17^	/	/	No	50.8 (5–228)			3/26 (11.5)			
Moussata *et al*., 2007^25^	12 (57.1)	23/23 (100.0) 22/23 (95.7)	Yes (only polyps in the ileal mucosa of the pouch body are described)	Mean 5.4 +- 2.6 (1–11)	Mean 4.7+-3.3 years (1–14)		17/23 (74.0)	Mean size 5.2 mm +-3.4 mm; 3 polyps were > 10 mm.	LGD 16/17 (94.1); HGD 1/17 (5.9)	/
Groves *et al*., 2005^35^	35 (58.3)	/	Between pouch and above anastomosis ileum	6 years (1–17 years)	/	32.5 years (13–66 years)	34/60 (57%) of which 5 were > 10 mm / 11 were advance adenomas	Mean size 5 mm (1–40 mm)	/	Median number 4
Thompson-Fawcett *et al*., 2001^77^	/	20/33 (60.6) 18/20 (90.0)	Only pouch body	/	/	/	20/33 (60.0) adenomas	1–3 mm	/	Median 10 (1–100) Also lymphoid hyperplasia included

APC = adenomatous polyposis coli; FUP = follow up; HGD = high grade dysplasia; IRA = ileorectal anastomosis; LGD = low grade dysplasia

#### Cancer

Since the first case report of cancer arising in the ileal pouch of a FAP patient in 1994^6^, we have identified 45 (Table 6) cancers that have developed in FAP patients after primary IPAA. Of these, 30 were located in the pouch body and 15 in the anastomosis/rectal cuff. The time from surgery to cancer diagnosis was reported for 22 patients and ranged from 2.3^36^ to 33 years.^37^ The information about the interval since last follow-up was reported for only 15 patients. The shortest interval between normal endoscopic surveillance and cancer diagnosis was 9 months.^16^ Of the studies that reported the final outcome, 13 (28.9%) patients were alive at the last follow-up (range 8 months to 6 years) after surgical therapy and 9 patients died of disseminated cancer (1 month to 4 years after diagnosis), most despite an initial R0 resection.

**TABLE 6.** Cancer rate after primary ileal pouch anal anastomosis (IPAA)

**First author and publication date (ref.)**	**No of patients**	**Age at cancer diagnosis (years)**	**Time to cancer (years)**	**Interval since last surveillance endoscopy and findings**	**Endoscopic findings at diagnosis**	**Location**	**Staging of cancer and status**
Aelvoet *et al*., 2023^55^	3/111 (2.7%)	/	/	/	/	/	Pouch excision
Pasquer *et al*., 2021^58^	1/92 (1.1)	30	/	1 month		Pouch body	Endoscopic resection
Ganschow *et al*., 2018^61^	1	/	27	/	/	Pouch body	Resection and reconstruction of a new pouch - alive
Walsh *et al*., 2016^63^	1	54	/	Regular annual surveillance	New endoscopy due to anemia and rectal blood loos	Anastomosis	T3N2Mx, resection and ileostomy, alive during last FUP.
Wasmuth *et al.*, 2013^67^	1	/	11	/	/	Rectal cuff	Resection and ileostomy - alive
Boostrom *et al.*, 2013^66^	1	/	23.7	/	/	Pouch body	Transanal resection - alive
Ozdemir *et al.*, 2013^38^	4	/	Mucosectomy group; median 11.3 years (8.3–22) Without mucosecomy; 8 years	Regular annual surveillance	/	All ATZ	? 3 underwent APR - alive 1 transanal resection – died 4 years later dissemination
Makni *et al.*, 2012^69^	1	26	10	8 months	Polyps, LGD?	Pouch body?	Pouch excision – died 12 months later dissemination
Tonelli *et al.*, 2012^51^	2	29 58	10	12 months, normal 6 months, normal	? IIa + IIc polyp	Pouch body Pouch body	Excision with ileostomy, T3N0M0, died 6 months later dissemination/Excision with ileostomy, T2N0M0, alive after 56 month FUP
voon Roon *et al.*, 2011^70^	1	/	13	/	/	Pouch body	Excision of a pouch – died 2 years of disseminated disease
Banasiewicz *et al.*, 2011^33^	5	/	/	/	/	Pouch body	/
Ault *et al.*, 2009^71^	2	61 50	11 10	6, normal /	Pain and blood per rectum, 3 cm mass/Sacral pain, bleeding ulcer	Pouch body / Pouch body	T2N1Mx, died of AMI prior treatment / Metastatic disease, chemotherapy
Tajika *et al.*, 2009^83^	2	55 68	8.6 20	9 months, normal No FUP	30×25 mm cancer / Polyposis and 25 × 25 mm polyp	Pouch body/Kock’s pouch body	T4N2M0 – died 1 year later T3N?M? – died (MDS)
Lee *et al.*, 2008^71^	1	/	7	/	Ulcerating tumor	Pouch body	T4N1M0, APR ileostomy. Developed metastases 2 years later.
Friederich *et al.*, 2008^32^	4	35 37 32 36	14 10.2 16.4 6.2	4.4 years, normal 2.1 years, normal No control (symptoms) 0.6 years, Tubullovilous HGD	/	All pouch body	Dukes C Dukes B Dukes B Dukes B
Linehan *et al.*, 2007^72^	1	40	10	/	Pelvic pain, discharge	Pouch body (patient had ileostomy but pouch was left in situ)	Excision. At last FUP patient was well.
Ulas *et al.*, 2006^74^	1	/	9	/	/	Anastomosis	Dukes B, APR, metachronous cancer after 1 year
Campos *et al.*, 2005^19^	1	/	12	No FUP	Presented with rectal bleeding	Pouch body	T2N0Mx, APR and ileostomy, patient well at 6 years FUP.
Vroueraets *et al.*, 2004^75^	2	48 36	9 10	5 years normal, then 2 and 1 years (both multiple LGD adenomas refused surgery) / Regular FUP every 2 years	Presented after 1 year with rectal bleeding /Normal. Routine biopsies at subsequent FUP revealed adenoca.	Anastomosis Anastomosis	T2N0M0, APR, alive 1 year later / T4N0M0, APR, alive 8 months later
Cherki *et al.*, 2003^76^	1	35	3.5	1.5 years	/	Pouch body	T3N1M1, resection with ileostomy, died 1 month later
Ooi *et al.*, 2003^36^	2	36 /	2 years 3 months 8 years	/ /	Symptoms of anal bleeding/ /	Anastomosis Anastomosis	T3NOMO, APR, ileostomy, died 2.5 years later dissemination / T2N0M0, transanal excision with ileostomy (refused APR), died 4 years later, dissemination
Brown *et al.*, 2001^78^	1	44	7 years 4 months	Under FUP every 6 months	/	Anastomosis	/
Vuilleumier *et al.*, 2000^79^	1	38	7	No FUP	/	Anastomosis	Resection with ileostomy – died 12 months later dissemination
Palkar *et al.*, 1997^15^	1	39	4.7	3 months	?	Pouch body	T4NOM? - alive
Kim *et al.*, 1997^15^	1	/	/	/	/	Pouch body?	/
Bassuini *et al.*, 1996^80^	1	31	3	No FUP	/	Pouch body	/
Von Herbay *et al.*, 1996^14^	1	33	8			Anastomosis	T1N0M0
Hoehner *et al.*, 1994^7^	1	34	20	/	/	Anastomosis	/

The data from these cases has been drawn from reviews by Tajika and Smith as full-text of the papers were not accessible.

FUP = follow up; HGD = high grade dysplasia; LGD = low grade dysplasia

#### Hand-sewn vs. stapled IPAA

Six studies (Supplementary Table 3) compared the rates of adenoma development at the anastomosis between hand-sewn and stapled techniques. The incidence of adenoma was lower for hand-sewn anastomosis, ranging from 0 to 33%, and for stapled anastomosis, ranging from 33.9 to 57%. The 10-year cumulative risk of adenoma development is 20–22.6% for hand-sewn anastomosis and 51.1–64% for stapled anastomosis.

#### Risk factors for adenoma development after primary IPAA

Nine studies analysed risk factors for adenoma development (Supplementary Table 4). None of the seven studies found a genotype-phenotype association. There was no association between colon adenoma burden at the time of surgery and subsequent development of pouch adenomas in three out of four studies. In the only positive study, none of the patients with < 200 colon polyps developed pouch adenomas, whereas almost half of the patients with > 1000 colon polyps later developed later pouch adenomas. Three studies have identified age of the pouch as a risk factor, while three others found no association between time since surgery and the rate of pouch adenomas. An association between the Spigelman score and the development of pouch adenomas was not confirmed. One study identified the presence of gastric adenomas as an independent risk factor for the development of pouch adenomas.

## Discussion

Using a systematic approach, we identified a wide range of reported adenoma and cancer rates in the rectal remnant, pouch body, at IPAA and in the neoterminal ileum. The wide range in adenoma rates is probably partly due to the wide range of included studies in terms of year of publication. The equipment and quality of optical diagnosis has improved considerably in recent years, allowing better detection of adenomas and more precise examination of the pouch and rectal remnants. In addition, the risk stratification of patients at the time of surgery has also improved, allowing patients with a more aggressive phenotype to undergo primary restorative proctocolectomy while primary IRA can still be offered to patients with an attenuated phenotype or low rectal disease burden. Indeed, in the largest study of four European national polyposis registries, the cumulative risk of cancer in the rectal remnant (CRR) was 10% in patients operated in the ‘pre-pouch’ period and only 2% in those who were operated in the ‘pouch period.^24^ Similar findings have been reported from the USA^38^ where 8 patients operated before 1983 (12.9%) were diagnosed with CRR compared to none of those operated after 1983 when pouch surgery was introduced at the Cleveland Clinic. Recently published data from two Japanese studies reporting an overall CRR rate of 30%^25^ – 37%^20^ must be interpreted with caution as carcinoma *in situ* was also included in the definition of cancer in their cohorts. The risk of metachronous cancer after IRA has been recognised early and these patients have been advised to undergo regular surveillance of the rectal remnant. Traditionally, surveillance was recommended every 3 to 12 months. This recommendation has been maintained ever since and can be also found in the recently published international guidelines (Table 1). The French national guidelines published in 2005^39^ are the only ones to include the genotype information, as they recommend more frequent surveillance if the pathogenic variant is located between codons 1250–1500. However, they were published in 2005.

The main obstacle to refining recommendations for endoscopic surveillance is the lack of high-quality, prospective data. Unfortunately, we have not found a single randomised trial that has compared different surveillance strategies or aimed to identify factors that would allow risk stratification. Members of the International Society for Gastrointestinal Hereditary Tumors (InSiGHT)^40^ proposed a staging system^41^ and stage-specific interventions for patients with intact colon and those with IRA, but unfortunately no effort has been made to validate this staging system. Data on endoscopic treatment modalities are even more descriptive. In fact, in five international recommendations (Table 1), only Vasen *et al*.^7^ recommended endoscopic removal of all polyps with dysplasia or those larger than 5 mm. Endoscopic management of these patients has therefore been influenced by expert groups. Unfortunately, preferred methods of endoscopic management were rarely described in the reviewed studies. Maehata *et al.*^20^ recommend removal of all polyps larger than 8 mm. A descriptive study with a small sample size (n = 6)^42^ showed that large-scale cold snare polypectomy can effectively reduce the polyp burden in the rectal remnant even in cases of very high polyp numbers. The mean number of polyps removed was 78.5 (30–155). During the follow-up (mean 10.7 months), none of the patients developed rectal cancer and there were no complications related to polypectomy. This is in contrast to another study from the USA^30^, which advocates the use of ablative therapy with argon plasma coagulation. A similar practice was supported by a study published in France in 2007.^17^ National French guidelines published in 2005 recommend ablation with APC for small polyps (a few millimetres) and mucosectomy for larger polyps.^39^

**FIGURE 2.**
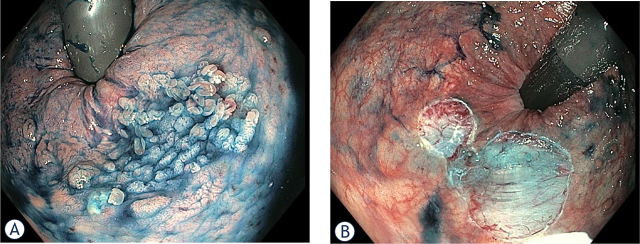
Surveillance endoscopy in a 48-year old patient with FAP after colectomy with IRA revealed 18 m LST-G **(A)**. After submucosal injection with gelofusine, indigo carmine and adrenaline, piecemeal endoscopic mucosal resection (pEMR) **(B)** was performed.

Improvements in endoscopic resection techniques have also been applied to the treatment of large lesions in the rectal remnant. Recently two reports, both from Japan^43,44^, have been published of successful endoscopic submucosal dissection (ESD) of 75 mm Is + IIa adenoma and residual adenoma at the IRA. In our endoscopy unit (Hospital Clinic, Barcelona) we also perform advanced endoscopic resection techniques. Figure 2 (A and B) shows a recent endoscopic mucosal resection (EMR) of an 18mm laterally spreading tumour granular type (LST-G) in the rectal remnant of a patient with FAP.

There is little data on the use of advanced imaging techniques. The study from St. Mark’s hospital in London^45^ showed no benefit of dye-based chromoendoscopy to detect additional adenomas in the rectal remnant. The European Society of Gastrointestinal Endoscopy (ESGE) guidelines^46^ published in 2014 did not recommend the use of advanced endoscopic imaging in patients with FAP, but did not specifically differentiate between the patients with intact colon and those after surgery. On the other hand, the French Society of Endoscopy^39^ recommended the use of dye-based chromoendoscopy with indigo carmine. We believe that use of dye-based chromoendoscopy in these patients does not increase the detection of clinically relevant lesions and it is not routinely performed in our unit. Considering the data on a cumulative risk of 57% for CRR 30 years after surgery^20^ and the fact that adenoma development in the rectal remnant is an inevitable event^16^, regular endoscopic surveillance is mandatory. Our recommendations are in line with other guidelines and our patients are recommended annual endoscopic surveillance, despite alarming data from an early study published in 2001^5^ from four European registries in which 75% of patients with CRR had a negative rectoscopy within 12 months and 35% within 6 months prior to diagnosis of CRR. There was no information on the endoscopy equipment used for surveillance. We believe that the high rates of negative rectoscopies prior to cancer diagnosis may – to some extent - be influenced by the quality of endoscopy, which has been limited by the technical aspects of the equipment used in the past. This problem needs to be addressed again in the light of developments in endoscopic equipment.

When restorative proctocolectomy with IPAA was first described in 1978^47^, it was believed that this operation would eliminate the risk of colorectal cancer in patients with FAP. However, a few years later, as the first pouches began to age, case reports of cancers arising in the pouch began to appear in the literature.^6^ Since then, reports have become more frequent and we have identified 45 cases of cancer after primary IPAA, of which 26 arose in the ileal mucosa of the pouch body and 15 at the anastomosis. Furthermore, we now know that cancer can develop even after mucosectomy down to the dentate line^48^, because even after removal of all visible rectal mucosa, some microscopic rectal columnar epithelium remains at the ATZ.^49^ In the study from the Heidelberg Polyposis Registry with 100 patients^50^, rectal residual mucosa (defined as visible mucosa or detected by histology from blinded biopsies) was found in 42 (84%) cases after stapled and in 21 (42%) cases after hand-sewn anastomosis.

Researchers from Japan^16^ found a 70% incidence of adenomas in the pouch body with one of the longest follow-up periods reported to date (> 20 years). Similarly, in a study from France, 74% of patients had at least one adenoma in the pouch, but with a mean follow-up of only 5.4 years. In contrast, one study found that isolated rectal cuff adenomas were more common than isolated pouch adenomas (49.1% *vs.* 6.8%), while 18.7% of patients had both pouch and rectal cuff adenomas. Cumulative 5-year, 10-year and 20-year risks for pouch adenomas were 32%, 52% and 68% in the Japanese study^16^, a slightly lower 5-year cumulative risk but a similarly high 10-year risk was observed in a Dutch study^31^; 16% and 42%, but the authors of this paper did not specifically define the exact location of the adenomas. The authors also reported a 10-year cumulative risk of developing precancerous adenomas of 12.8%.

On the other hand, the adenoma rates – at least in the stapled group - seem to be higher in the studies that only looked at the anastomosis and compared hand-sewn with stapled: 0–33% *vs.* 33.9–57%. In view of these figures, it is essential that patients with primary IPAA also undergo regular endoscopic surveillance. Particular attention should be paid to the rectal cuff and anastomosis, and the pouch should be examined in both forward and retroflexed position.

International guidelines most commonly recommend annual endoscopy examination, whereas ASCO guidelines^9^ advocate ‘case-by-case’ interval allocation. In 11 of only 12 studies that described a surveillance protocol, an interval of 12 months was recommended except in Brazil where endoscopy of the pouch was recommended every 2 years.

Interestingly, in the Netherlands pouch endoscopy was recommended every 1 to 3 years in the late 1990s but in 2001 the protocol was changed to annual endoscopic surveillance regardless of the anastomotic technique (hand-sewn or stapled).

One of the main concerns is the short interval (< 1 year) between the last normal endoscopy and the cancer diagnosis and the aggressive course of the disease despite an initial R0 resection (Supplementary Table 4). It is not entirely clear whether the adenoma-carcinoma sequence is faster in the ileal mucosa compared with the colon and rectum, or whether “negative” endoscopies prior to cancer diagnosis could be explained by the poor quality of pouch endoscopy. Chromoendoscopy improves the detection of diminutive adenomas^31^ and lymphoid hyperplastic nodules^45^, but its use is discouraged^33,35^ for the same reasons as in the examination of rectal remnants – increased of detection of clinically irrelevant polyps. Endoscopy should be performed with a gastroscope or paediatric colonoscope, as stricture can occur at the anastomosis, especially after hand suturing.

**FIGURE 3.**
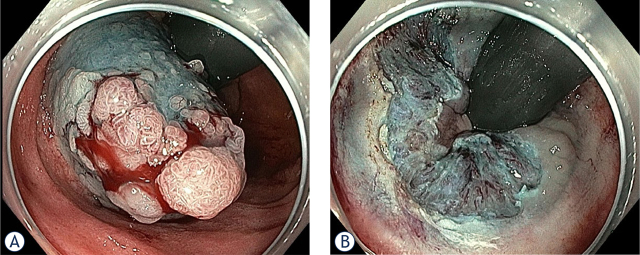
Surveillance endoscopy in a 49-year old patient with FAP after proctocolectomy with IPAA revealed 25 mm LST-G mixed type lesion in the rectal cuff. Lesion was spreading from the anastomosis to the dentate line. Patient had undergone surgery five years earlier and did not show up for endoscopy follow-up since then **(A)**. Lesion was removed with pEMR **(B)**.

There are no official recommendations for endoscopic management of FAP patients after IPAA. We have found considerable heterogeneity in local practice. Italian authors recommend resection of all adenomas > 3 mm.^51^ On the contrary, ablation with argon plasma coagulation is the preferred resection technique in a French study.^17^ Ablative techniques were also supported by the study from the Mayo Clinic.^30^ In a small descriptive cohort of only 5 patients^42^, large-scale cold snare polypectomy with a mean of 110.6 (30–342) resected polyps demonstrated the efficacy of cold snare in controlling large polyp burden (> 30 polyps) with no reported polypectomy related complication. In our unit we do not use nor encourage use of argon plasma coagulation. We recommend resection of all polyps > 3 mm. Advanced resection techniques, when performed in the tertiary centres, may be a viable alternative prior to surgical resection. A case report of successful *en bloc* ESD of a 15 mm ‘non-lifting’ HGD adenoma in the ileal pouch has recently been published.^52^ Figure 3 (A and B) shows an EMR of 25 mm LST in a patient with FAP after IPAA. The polyp was located in the rectal cuff and extended from the anastomosis to the dentate line. The procedure was performed at our Endoscopy Unit. It should be emphasised that the wall of the ileum is very thin and special care must be taken when resecting larger lesions.

Although there is no randomised trial comparing different endoscopic surveillance intervals, it is unlikely that prospective data will be available in the future. The main reason is ethical issue, as these patients are at increased risk of colorectal cancer. However, with the introduction of high quality colonoscopy and improvements in endoscopy technique, a ‘negative’ endoscopy before cancer diagnosis should become highly unlikely if not impossible.

## Supplementary Material

Supplementary Material Details
