# Supplementary material for: Endoscopic management of patients with familial adenomatous polyposis after prophylactic colectomy or restorative proctocolectomy – systematic review of the literature
Source: Radiol Oncol. 2024 Jun 11;58(2):153–69. doi: 10.2478/raon-2024-0029 (PMC11202397; doi:10.2478/raon-2024-0029)
Supplement: Supplementary file 1 — Supplementary Material Details [file raon-2024-0029-sm.pdf]

sciendo Radiology and Oncology | Ljubljana | Slovenia | www.radioloncol.com RADIOLOGY AND ONCOLOGY

Endoscopic management of patients with familial adenomatous polyposis after prophylactic colectomy or restorative proctocolectomy - systematic review of the literature

Aleksandar Gavric, Liseth Rivero Sanchez, Angelo Brunori, Raquel Bravo, Francesc Balaguer, Maria Pellisé

doi: 10.2478/raon-2024-0029

SUPPLEMENTARY TABLE 1. Treatment modalities of adenomas, rate of secondary proctectomy and adenoma development rate after ileorectal anastomosis (IRA)

First author and publication date	Secondary proctectomy for worsening polyposis; n/(%)	Cumulative risk for proctectomy	Secondary proctectomy with IPAA ir IAA for rectal remnant cancer; n/(%)	Years since primary IRA to surgery for all cases	Years since primary IRA to surgery for rectal cancer	Endoscopic resection of adenomas	Endoscopic resection of rectal remnant cancer; n (%)	Cumulative risk for adenomas	Adenoma proportion
Tajika *et al.*, 2019[16]	/	/	/	/	/	/	/	85% at 5 years and 100% at 10 years.	14 / 14 (100)
Patel *et al.*, 2016[42]	0/6	/	0/6	/	/	Large scale cold snaring. Mean of resected polyps 78.5 (30–155)	/	/	/
Maehata *et al.*, 2015[20]	/	/	5/27 (18.5)	/	/	16/27 (59.3) Removed in total	6/27 (22.2) (All were TisN0M0)	/	/

First author and publication date	Secondary proctectomy for worsening polyposis; n/(%)	Cumulative risk for proctectomy	Secondary proctectomy with IPAA ir IAA for rectal remnant cancer; n/(%)	Years since primary IRA to surgery for all cases	Years since primary IRA to surgery for rectal cancer	Endoscopic resection of adenomas	Endoscopic resection of rectal remnant cancer; n (%)	Cumulative risk for adenomas	Adenoma proportion
						178 large adenomas.			
Pommaret *et al.*, 2013[35]	/	/	/	/	/	1 advance adenoma - EMR	/	/	12/13 (92.3)
Koskenvuo *et al.*, 2013[22]	17/140 (12.1)	/	17/140 (12.1)	/	/	/	/	/	/
Booij *et al.*, 2010[18]	5/34 (14.7)	/	2/34 (5.9)	Mean 182 months (85–277)	/	/	/	/	/
Sinha *et al.*, 2010[26]	77/427 (18.0)	/	48/427 (11.3)	Median 15 (7–25) for all causes	/	/	/	/	/
Nieuwenhuis *et al.*, 2009[27*]	Group 1: 4/58 (6.8%)<br>Group 2: 135/362 (37.0)<br>Group 3: 29/55 (53.0%)	Cumulative risk 20 years after surgery:<br>Group 1: 10%<br>Group 2: 39%<br>Group 3: 61%[%]	Group 1: 1/58 (1.7)<br>Group 2: 29/362 (8.0)<br>Group 3: 4/55 (7.3)	/	/	/	/	/	/
Yamaguchi *et al.*, 2009[25]	/	/	9/59 (15.3)<br>APR; 2/59 (3.4)	/	8.8 years (1.3 – 23.3)	/	EMR; 5/59 (8.5)<br>TAMIS; 1/59 (1.7)	/	/
Gleeson *et al.*, 2008[30]	/	/	2/16 ()	/	13 and 12 years (patients had no prior endoscopic surveillance)	/	/	/	16/16 (100)
Bullow *et al.*, 2008[24]	163/776 (21.0)	/	/	/	Median 12 years (1–40 years)	/	/	/	/
Campos *et al.*, 2008[19]	/	/	6 (16.7%)<br>Local resection 2<br>Proctectomy 3	/	34 months – 132 months	/	/	/	/

First author and publication date	Secondary proctectomy for worsening polyposis; n/(%)	Cumulative risk for proctectomy	Secondary proctectomy with IPAA ir IAA for rectal remnant cancer; n/(%)	Years since primary IRA to surgery for all cases	Years since primary IRA to surgery for rectal cancer	Endoscopic resection of adenomas	Endoscopic resection of rectal remnant cancer; n (%)	Cumulative risk for adenomas	Adenoma proportion
			Metastatic disease not resected						
Moussata *et al*., 2007[17]	1/21 (4.7)	/	0 (0.0)	29 years since IRA	/	Before 1995 all polyps in ileal mucosa above IRA were treated with Yag laser and after 1995 with apc.	/	/	10/21 (48.0) of which 2 were advanced adenomas. In the ileal mucosa above IRA.
Church *et al*., 2003[38]	15/197 (7.6)	/	8/197 (4.1)	/	/	/	/	/	/
Church *et al*., 2001[15]	Group 1: 5/74 (6.8)<br>Group 2: 2/54 (3.7)<br>Group 3: 13/37 (35.0)[$]	/	Group 1: 0/74<br>Group 2: 2/54 (3.7)<br>Group 3: 4/37 (10.8)[$]	/	/	/	/	/	/
Bertario *et al*., 2000[73]	/	/	/	/	/	/	/	/	/
Jenner *et al*., 1998[21]	/	/	7/55 (12.7);<br>IPAA: 4<br>APR: 2<br>Ultra low IRA: 1	/	(4–28 years)	/	/	/	/

[*]Colonic phenotype divided in 3 groups. Group 1: < 100 polyps and mutation in codons 1–157, 312–412 and 1596–2843; Group 2: hundreds of polyps and mutation in codons 158–311, 413–1249 and 1465–1595; Group 3: thousands of polyps and mutation in codon 1250–1464. %-rate of proctectomies for all reasons are given. [$] Group 1: < 5 adenomas in the rectum at the time of surgery; Group 2: 6–19; Group 3 > 20 adenomas. Causes for secondary proctectomies are not stratified by indication.

SUPPLEMENTARY TABLE 2. Overview of the risk factors for progressive rectal remnant phenotype after ileorectal anastomosis (IRA)

First author and publication date (ref.)	APC pathogenic variant	Colonic / Rectal phenotype before surgery	Time since surgery	Age of the patient (years)	Period of surgery	Presence of duodenal adenomas	Rectal remnant phenotype during surveillance	Congenital hypertrophy of the retinal pigment	Cancer in the resected colon at the time of initial surgery
Maehata *et al.*, 2015[20]	No	No	/	/	/	No	No	Independent risk factor on multivariate analysis	
Koskenvuo *et al.*, 2013[22]	/	/	Cumulative risk for cancer 9% 30 years after surgery	/	/		/		
Sinha *et al.*, 2010[26] *Risk factors for rectal survival after IRA	Codon 1250–1450); HR 3.91 (1.45–10.51), P = 0.007	Colonic count > 500; HR 2.18 (1.24–3.82), P = 0.006 Rectal count > 20; HR 30.99 (9.57–100.32), P < 0.001	Age < 25 at the surgery; HR 1.99 (1.17–3.37), P = 0.011.		/	/	/	/	
Yamaguchi *et al.*, 2009[25]		Dense type (> 2000 polyps) in 60% *vs.* 24 % without					Dense type (> 20 polyps) - 80% developed cancer and 25 % without dense type developed cancer		
Nieuwenhuis *et al.*, 2009[27]	/	Severe colonic phenotyp (thousands of polyps and mutation in codon 1250–1464) had the highest cumulative risk for secondary proctectomy due to polyposis or cancer.							

First author and publication date (ref.)	APC pathogenic variant	Colonic / Rectal phenotype before surgery	Time since surgery	Age of the patient (years)	Period of surgery	Presence of duodenal adenomas	Rectal remnant phenotype during surveillance	Congenital hypertrophy of the retinal pigment	Cancer in the resected colon at the time of initial surgery
Bullow *et al.*, 2008[24]	/	/	/	/	Cumulative risk of rectal cancer was lower in female operated in pouch period (after 1990)		/		
Campos *et al.*, 2008[19]	/		/	45.8 (36.6 without metachronous rectal cancer)	/	/	/	/	5/6 of patients with metachronous rectal cancer had cancer in the colon during the primary surgery
Valanzano *et al.*, 2007[28]	All 3 patients (of total 25) with carpeting polyposis in rectal remnant had mutation at the codon 1309 (Exon 15) - only descriptive.	Significant difference between polyps at FUP was seen when cut-off of 10 polyps in rectal remnant used. Mean No of polyps/year/patient: 0.67 (< 5 polyps in rectum at surgery) / 1.62 (6–9) and 9.29 (> 10)	/	/	/	/	/	/	/
Church *et al.*, 2003[38]	/	/	/	/	8 (12.9) patients developed rectal cancer operated < 1983 and 0 (0.0) operated > 1983	/	/	/	
Church *et al.*, 2001[15]		> 20 rectal polyps *vs.* < 20 rectal polyps 4/74 (5.4) *vs.* 2/139 (1.4) later rectal							

First author and publication date (ref.)	APC pathogenic variant	Colonic / Rectal phenotype before surgery	Time since surgery	Age of the patient (years)	Period of surgery	Presence of duodenal adenomas	Rectal remnant phenotype during surveillance	Congenital hypertrophy of the retinal pigment	Cancer in the resected colon at the time of initial surgery
		cancer							
Bertario *et al*., 2000[23]	Yes (HR 4.4 (1.3–15.0) for mutation between codons 1250–1464)*								Yes (HR 3.2 [1.1–9.8])*

*On the multivariate analysis. #On the univariate analysis also >30 polyps in the rectal remnant (HR 4.6 [1.2–17.9])

SUPPLEMENTARY TABLE 3. Studies comparing hand-sewn vs. stapled ileal pouch anal anastomosis (IPAA)

Study	Man proportion	Median follow-up (years)	Time from surgery to first adenomas	Age at surgery	Rate of adenomas in stapled anastomosis	Rate of adenomas in hand-sewn anastomosis	*P* value	Histology of adenomas	Size of Adenomas
Goldstein *et al.*, 2015[64*]		Mean 11.6 years +- 14.6 years		Mean 30.8 +-10.8 years	24/42 (57.0)	5/15 (33%)	0.25	/	/
Ganschow *et al.*, 2015[50]	51 (51.0)	44.8 months (stapled); 146.1 months (handsewn)	/	28.5 years (14–56) stapled; 29.3 (7–58) handsewn.	21/50 (42.0)	10/50 (20.0)	0.02	/	/
Zahid *et al.*, 2015[65]					12/22 (55.0)	0/5 (0.0)	0.047		
Ozdemir *et al.*, 2013[37]	140 (53.8)	Median 95 +- 70; 155 +-99	Median 40 months (5–244) stapled; 107 months (19–331) handsewn.	/	59/174 (33.9)	18/86 (20.9)	0.03	Hand-sewn: LGD 12/14; HGD 2/14 Stapled: LGD 22/26, HGD 4/26	Hand-sewn: < 10 mm 12, 10–20 mm 3; 20–30 mm 3 Stapled < 10 mm 45, 10–20 mm 10, 20–30 mm 4.
von Roon *et al.*, 2011[70]	69	Median 7.2 years (1.9–20.6); 11.3 years (0.7–24.5)	6.5 years (stapled); 10.1 years (hand-sewn)	31 (13–58) stapled; 32 (14–62) handsewn	24/44 (54.5%); Cumulative 10-years risk 51.1%	21/76 (27.6%); Cumulative 10-years risk 22.6%	*P* < 0.001 (for cumulative risk)		9 adenomas > 10 mm: 5 in stapled group > 10mm; 3 in handsewn; 1 the techniquenot documented.
Friederich *et al.*, 2008[31]		Mean 7.9 (0.4–20.3)		Mean 30.0 (10.0–62.6)	Cumulative 10-year risk 64 %	Cumulative 10-year risk 20 %	*P* = 0.0004		

SUPPLEMENTARY TABLE 4. Overview of the risk factors for adenoma development after primary ileal pouch anal anastomosis (IPAA)

First author and publication date	APC mutation site	Colonic phenotype	Time since surgery	Age of the patient	Presence of duodenal adenomas or severity of polyposis	Spigelman score	Presence of desmoid tumor	Sex	Presence of gastric adenomas
Ganschow *et al*., 2018[61]	No	/	Yes (Mean 13.9; SD 6.9 vs. 8.1; 6.2) (P < 0.0001)	Age < 18[#] years at surgery (P = 0.0173)	Yes (On univariate analysis but not on multivariate)	/	No	Man[#] (P = 0.0017)	Yes[#] (P = 0.0019)
Kariv *et al*., 2017[62]	Mean No. of pouch adenomas higher among carriers of exon 15 *vs*. exons 1–14 (Mean No. of polyps 2.2 +- 3.7 vs. 0.5 +-2.4) and cuff adenomas (0.8 +- 1.3 vs. 0.3 +-0.8)[&]								
Goldstein *et al*., 2015[64*]	/	No	No		Yes (P = 0.001)	/	No		
Pommaret *et al*., 2013[35]	/				Yes (OR, 4.35; P = 0.011)				
Tonelli *et al*., 2012[51]	No	Yes (< 200 polyps did not develop pouch adenomas; > 1000 colonic polyps; 46% developed pouch adenomas)	No	Yes (26.2 ± 8.4 *vs*. 32.6 ± 11.9 years; P = 0.02)	Yes	No			
von Roon *et al*., 2011[70+]	No	No	/	Age > 40 at surgery	/	/	/	/	/
Moussata *et al*., 2007[17]	No	/	No	No	/	No	/	No	/
Groves	No	No	Yes[#]	Yes[#]	No	/	/	No	/

First author and publication date	APC mutation site	Colonic phenotype	Time since surgery	Age of the patient	Presence of duodenal adenomas or severity of polyposis	Spigelman score	Presence of desmoid tumor	Sex	Presence of gastric adenomas
et al., 2005[34]			Median 7 (2–1/9 *vs.* 4 (1–14);<br>(P < 0.05)	Median 42 (19–72) vs. 35.5 (16–64); (P < 0.02)					
Thompson-Fawcett *et al.*, 2001[74]	No		Yes<br>(P < 0.001)		No				

*Presence of pouch adenomas was significantly correlated with the presence of cuff adenomas (P = 0.043). # On multivariate analysis; + Risk factors for ATZ adenomas; & Difference was not statistically significant

SUPPLEMENTARY TABLE 5. Treatment of polyps after primary ileal pouch anal anastomosis (IPAA)

First author and publication date (ref.)	Endoscopic therapy of polyps	Endoscopic surveillance interval
Tajika *et al.*, 2019[16]		IRA – every 6 months<br>IPAA – every 12 months
Ganschow *et al.*, 2018[61]		Every 12 months
Patel *et al.*, 2016[42]	Large scale cold snaring.<br>Mean number of resected polyps: 110.6 (30–342)	
Goldstein *et al.*, 2015[64*]	All adenomas were resected endoscopically or transannally without need for pouch resection	
Zahid *et al.*, 2015[19]		
Kennedy *et al.*, 2014[66]	All polyps were treated by endoscopic resection	
Ozdemir *et al.*, 2013[37]	Polyps at the ATZ:<br>- Polypectomy 68<br>- Excision of ATZ 5<br>- APR 3	Every 12 months
Pommaret *et al.*, 2013[35]	4 advanced adenomas - TAR<br>7 advanced adenomas - polypectomy<br>3 advanced adenomas - 3 EMR<br>1 advance adenoma - surgical resection of the pouch	
Wasmuth *et al.*, 2013 [67]		Every 12 months
Tonelli *et al.*,	3/69 (4.3) Open surgical resection	Every 12 months

First author and publication date (ref.)	Endoscopic therapy of polyps	Endoscopic surveillance interval
2012[51]	4/69 (5.8) Transanal resection<br>13/69 (18.8) Ablation with diathermia	
Banasiewicz *et al*., 2011[32]		Every 12 months
voon Roon *et al*., 2011[70+]	All polyps were resected endoscopically	Every 12 months (3 to 6 months if clinically indicated)
Boostrom *et al*., 2013[75]		Every 12 months
Friederich *et al*., 2008[31]	Thermal ablation with argon plasma coagulation (55%), polypectomy (7%)	It was every 36 months but changed to 12 months
Campos *et al*., 2008[19]		Every 24 months
Moussata *et al*., 2007[17]	All visible polyps were treated with Yag laser < 1995 and with AP after 1995. Info only for polyps in the ileal mucosa in the pouch body.<br>1 polyp above anal verge required surgery. Other with APC (in one patient perforation and later surgery)	Every 12 months
Ooi *et al*., 2003[36]	/	Every 12 months, cautery in case of small polyps and reassessment in 6–12 months

EMR = endoscopic mucosal resection; IRA = ileorectal anastomosis; TAR = transanal resection
